# Regional Variability in the Access to Cardiac Rehabilitation in Poland

**DOI:** 10.3390/healthcare8040468

**Published:** 2020-11-09

**Authors:** Maciej Jankowiak, Justyna Rój

**Affiliations:** 1 Department of Organization and Healthcare Management, Poznan University of Medical Sciences, ul. Przybyszewskiego 39, 60-356 Poznań, Poland; mjankowiak@ump.edu.pl; 2 Department of Operational Research and Mathematical Economics, The Poznań University of Economics and Business, Al. Niepodległości 10, 61-875 Poznań, Poland

**Keywords:** Perkal’s method, cardiological rehabilitation, cardiovascular diseases, access, equity

## Abstract

Equitable access to cardiological rehabilitation services is one of the important elements in the effectiveness of the treatment of cardiovascular diseases as cardiological rehabilitation is an important part of circulatory system disease prevention and treatment. However, in many countries among others, Poland suffers from the underutilization of cardiac rehabilitation services. Cardiovascular diseases are the worldwide number one cause of mortality, morbidity, and disability and are responsible for the substantial increase in health care costs. Thus, the aim of the research was the analysis of geographical accessibility to cardiac rehabilitation services in Poland. Perkal’s method was employed in this research. The conducted research allowed to recognize the regional variation, but also made it possible to classify Polish voivodeships in terms of the level of availability achieved. This enables the identification of voivodeships that provide a good, or even very good, access to cardiology rehabilitation services and those characterized by low, or very low access. It was found that there was a slight regional variability in the access to cardiological rehabilitation services. However, the sufficient development of a rehabilitation infrastructure has been also recognized.

## 1. Introduction

Cardiovascular diseases (CVD) are the most-prevalent noncommunicable diseases and the number one cause of mortality, morbidity, and disability, therefore it substantially increases health care costs [1,2]. In the group of CVD, coronary artery disease (CAD) was solely one of the main causes of death in 2016 and in Europe, it was responsible for half of the deaths from heart diseases and 20% of the total number of deaths [3]. Coronary artery disease (CAD) is a broad term and covers, regardless of the factors causing it, all coronary artery disease conditions [4]. It is divided into acute coronary syndromes (ACS) and chronic coronary syndromes (CCS) [5]. ACS consist of two types of acute myocardial infarction: ST-segment elevation myocardial infarction (STEMI) and non-ST-segment elevation myocardial infarction (NSTEMI) [6]. Unstable angina (UA), caused by myocardial ischemia without damage of the heart tissue, also belongs to ACS.

The problem is that deaths due to CAD in the European Union accounted for 18% of one million deaths that could be avoided thanks to effective public health, and 32% of 570,000 preventable deaths thanks to timely and effective treatment and secondary prevention [7,8]. Fortunately, it is noted that CAD mortality is declining as the age-standardized mortality rate decreased in 36 OECD (Organisation for Economic Co-operation and Development) countries by 42% in the years from 2000 to 2017 [9]. Among the reasons for the observed decrease in mortality due to coronary artery disease are not just the advances in the treatment of acute coronary syndromes, but also the advances in secondary prevention methods as the secondary prevention method of cardiovascular diseases could be considered as cardiological rehabilitation programs [10].

Cardiological rehabilitation is eloquently defined by the World Health Organization as “the coordinated sum of activities required to influence favorably the underlying cause of cardiovascular diseases as well as to provide the best possible physical, mental and social conditions, so that the patients may, by their own efforts, preserve, or resume optimal functioning in their community and through improved health behavior, slow, or reverse progression of disease” [2]. Integral to standard of care, cardiac rehabilitation plays a significant role in the management of heart diseases resulting in an improvement in the patients’ physical activity and quality of life, prolonging their survival and a decrease in healthcare costs [11]. Despite proven benefits through the secondary prevention of cardiovascular diseases (CVD) and the reduction in mortality, cardiac rehabilitation (CR) remains underutilized in cardiac patients [1]. Therefore, the problem of underutilization has been noticed and analyzed by many studies all over the world. Some of them have focused on the identification of factors influencing cardiac rehabilitation attendance in a particular country [12,13,14,15,16,17,18,19,20,21], on the different barriers to attending cardiac rehabilitation in a high-income country [22,23,24,25,26,27,28,29,30,31,32], and also according to gender [33,34] or more generally on the availability [35,36,37,38,39] as well as on the geographic aspect of CR utilization [40].

According to The European Society of Cardiology (ESC), The Polish Cardiac Society (PTK), The American Heart Association (AHA), The American Association of Cardiovascular and Pulmonary Rehabilitation (AACVPR), and the Agency for Health Care Policy and Research, a comprehensive cardiac rehabilitation program should contain specific core components in order to be efficient and successful. One of them is the equity in the access to a cardiac rehabilitation program.

Equity matters as it applies to fair opportunity for everyone to achieve their full health potential regardless of demographic, social, economic, or geographic status. Thus, any inequities in access to healthcare services may lead to a low level of population health [41]. Regarding accessibility to healthcare, four dimensions of access to healthcare have been recognized such as geographic accessibility, availability, financial accessibility, and acceptability [42]. It is also noted that geographic accessibility and availability are especially important as lower healthcare utilization would result in poorer healthcare outcomes [43,44]. This means that inadequate geographic accessibility could be one of the reasons for cardiac rehabilitation underutilization. 

The core purpose related to the research on the equity in the access to healthcare, is to identify regions where the provision of healthcare services should be higher, and regions that do not require such a high access to health benefits [44,45].

In this research, we focused on Poland as cardiovascular diseases has been one of the main causes of death in the Polish population for years [46], while access to CR in Poland remains alarmingly low [8]. However, the percentage of acute coronary syndrome (ACS) cases that were cardiology rehabilitated continued to increase in the years 2014–2019. In 2019, 5% of ACS cases were rehabilitated within 14 days from the date of admission, 25% within 60 days, and 28% within 90 days, which means that it was 2.3 percentage points (pp), then 9.0 pp, and 9.4 pp, respectively, more than in 2014. However, from 2014 to 2018, among the patients undergoing cardiac rehabilitation within 60 days from the date of admission due to ACS, the growing share of patients who underwent cardiac rehabilitation in daytime conditions and a decreasing percentage of patients undergoing inpatient cardiac rehabilitation were noticeable [8].

In Poland, a comprehensive care program after acute myocardial infarction (named in Polish “KOS-zawał”) has been in operation since 2017. The number of centers implementing the program was constantly growing and in December 2019, services were provided by 60 healthcare providers. In 2019, benefits under the program were granted to 19.6 thousand patients and the value of reimbursement of benefits amounted to PLN (the Polish currency-zloty) 197 million. Out of 9.4 thousand patients who received the above program in 2018, 76% had cardiac rehabilitation, which was 5.2 thousand. A total of 74% of patients undergoing cardiac rehabilitation started rehabilitation within 14 days from the date of discharge from the hospital due to a heart attack. Although there was a decline in the value of cost reimbursement made by the National Health Funds due to CAD, it was nearly PLN 2 billion in 2019 and was lower by PLN 529 million (21%) than in 2014, however the decrease in the cost of reimbursement was observed mainly in the case of services provided for unstable angina (a type ACS) and in the case of chronic CAD (by PLN 380 million and PLN 137 million, respectively). While in the case of myocardial infarctions, the value of reimbursement made by the National Health Funds increased by PLN 50 million (6%) and also in terms of the reimbursement value, hospital treatment dominated (93% in 2019). This is the result of the increase in the number of myocardial infarctions by 9% from 2014 to 2019 mainly due to the demographic structure of patients. Therefore, it is important to ensure equity in the access to cardiac rehabilitation in Poland [8].

Moreover, equal access to health services is one of the priorities of Polish health policy [47] and an important value of the healthcare system, as in the case of many healthcare systems over the world [48]. In addition, the establishment and development of CR services is essential for the most effective management of heart conditions [11,49]. Therefore, it is of high priority to verify the equity in the geographical access to CR in Poland.

Thus, the aim of the study was to assess the regional accessibility to cardiology rehabilitation centers in Poland. We assumed that potential (not actual) access to the health benefits, which is the result of the allocation of resources, was reflected in the usage of CR services, which is the outcome of the healthcare system. We formulated the following hypotheses that cardiology rehabilitation centers are distributed unequally among regions. To the best knowledge of the authors, this is the first research on the accessibility of cardiology rehabilitation in Poland across such a broad range with such methodology [50,51,52,53].

## 2. Materials and Methods

Data related to the number of cardiological rehabilitation centers in Poland in 2019 were derived from the Polish National Health Fund databases [54,55]. Centers were divided into two groups: the first group consisted of inpatient centers (hospital wards, where patients are admitted for all days and nights during the rehabilitation period), and the second group of outpatient centers (where patients return to their own homes after the end of each day of rehabilitation). The numbers of rehabilitation centers are presented separately for each of the 16 Polish districts (voivodeships) in Table 1.

The number of acute coronary syndromes (ACS) in Poland in 2019 was also acquired from the Polish National Health Fund databases. The ACS group consisted of the following ICD-10 (International Statistical Classification of Diseases and Related Health Problems, 10th Revision) classes: I20.0, I20.1, I21, I22, I23, and I24.0. The number of ACS broken down into 16 Polish districts are presented in Table 1.

In order to compare access to cardiac rehabilitation in each of the voivodeships, Perkal’s indicator was engaged. Perkal’s indicator is based on data aggregation using normalized variables. The general formula of Perkal’s indicator is given below [56,57].

W_k_ = 1/n Σ Z_ik_ (1)

where W_k_ is Perkal’s indicator for “k” voivodeship; n is the number of variables; and Z_ik_ is the normalized value of the “i” variable for “k” voivodeship.

Analyzed variables were divided into two classes. The first class, called “stimulants”, consisted of variables where an increasing value is associated with better access to rehabilitation. In our research, the class of “stimulants” consisted of two variables: the number of inpatient rehabilitation centers and the number of outpatient rehabilitation centers. The second class of variables (called “de-stimulants”) included variables that had a negative impact on access to cardiological rehabilitation. In this paper, one variable (the number of ACS) was classified as a “de-stimulant” due to the negative correlation between the number of patients in the early period after ACS and accessibility to rehabilitation.

Before aggregation variables need to be normalized, the process of normalization changes the raw variables to values without specific units of measurement that allows them to be aggregated together. Normalization formulas are different in the case of “stimulants” and “de-stimulants” due to their opposite impact on the assessed phenomenon. Normalization of variables belonging to the class of “stimulants” was calculated using the following formula [58]:

Z_ik_ = (X_ik_ − X_i mean_)/S_i_ (2)

where Z_ik_ is the normalized value of the “i” variable for the “k” district; X_ik_ is the raw value of the “i” variable for the “k” district; X_i mean_ is the mean value of the “i” variable for all districts; and S_i_ is the standard deviation of the “i” variable for all districts.

Normalization of “de-stimulants” was computed according to this formula [59]:

Z_ik_ = (X_i mean_ − X_ik_)/S_i_ (3)

where Z_ik_, X_ik_, X_i mean_, S_i_ are the symbols as above.

In our paper, two types of Perkal’s indicators were used. The first type of the indicator (in our paper called W^A^) aggregated two “stimulant” variables: the number of inpatient cardiology rehabilitation centers per 100,000 population (X_1_ before normalization, Z_1_ after normalization) and the number of outpatient cardiology rehabilitation centers per 100,000 population (X_2_ before normalization, Z_2_ after normalization). These variables were classified as “stimulants” because the increasing number of rehabilitation centers correlated with easier access to them. Perkal’s indicator W^A^ was calculated using the given formula below:

W^A^_k_ = ½ (Z_1k_ + Z_2k_) (4)

where W^A^_k_ is the value of the W^A^ indicator for the “k” district; Z_1k_ is the normalized value of the number of inpatient cardiology rehabilitation centers per 100,000 population in the “k” district; and Z_2k_ is the normalized value of the number of outpatient cardiology rehabilitation centers per 100,000 population in the “k” district.

The second type of Perkal’s indicator used in this paper (called W^B^), except the above-mentioned two “stimulants”, consisted of one “de-stimulant” variable: the number of ACS per 100,000 population (X_3_ before normalization, Z_3_ after normalization). This variable was involved in the W^B^ indicator in order to estimate the differences among Polish districts in demand for cardiological rehabilitation after ACS. This variable was treated as a “de-stimulant” because the increasing number of ACS leads to higher demand for cardiological rehabilitation and under the assumption of the constant number of rehabilitation centers, to limitations in accessibility to rehabilitation. Perkal’s indicator W^B^ was calculated using the given formula below:

W^B^_k_ = ⅓ (Z_1k_ + Z_2k_ + Z_3k_) (5)

where W^B^_k_ is the value of the W^B^ indicator for the “k” district; Z_1k_ is the normalized value of the number of inpatient cardiology rehabilitation centers per 100,000 population in the “k” district; Z_2k_ is the normalized value of the number of outpatient cardiology rehabilitation centers per 100,000 population in the “k” district; and Z_3k_ is the normalized value of the number of ACS per 100,000 population in the “k” district.

After calculation of the W^A^ and W^B^ indicator values, the below given qualitative classification of them based on their standard deviation was applied:

W_k_ > S_W_ is the above average value;

−S_W_ ≤ W_k_ ≥ S_W_ is the average value; and 

W_k_ < −S_W_ is the below average value,

where W_k_ is Perkal’s indicator value for the “k” district and S_W_ is the standard deviation of Perkal’s indicator values for all districts.

The range of values defining each of the three qualitative classes was different in the case of the W^A^ and W^B^ indicators and is given in the “Results” section. Calculation in this research was done using a free software spreadsheet (Trio Office).

## 3. Results

Results of the calculation of variables (X_1_, X_2_, X_3_) per 100,000 population and basic descriptive statistics (mean values and values of standard deviation) are presented in Table 2. The inter-district variation of the number of rehabilitation centers was high (the standard deviation represents above 40% of the mean value in the case of both inpatient and outpatient centers). The variation of the ACS numbers among districts was lower (the standard deviation comprises about 17% of the mean value).

Normalized values of variables Z_1_, Z_2_, and Z_3_ for all districts are presented in Table 3. These normalized values have been used during the construction of Perkal’s synthetic indicators W^A^ and W^B^.

Values of both types of Perkal’s indicators (W^A^ and W^B^) and their division into three qualitative classes (“below average”, “average”, and “above average”) are presented in Table 4. The values of the W^A^ indicator consisted of two variables: the number of inpatient cardiology rehabilitation centers and the number of outpatient cardiology rehabilitation centers were from −1.193 (the worst equipment with cardiological rehabilitation facilities in the Greater Poland voivodeship) to 0.995 (the best rehabilitation facilities in Lubelskie voivodeship). The standard deviation of the W^A^ indicator was 0.592, which means a high level of differences among districts.

**Figure 1:**
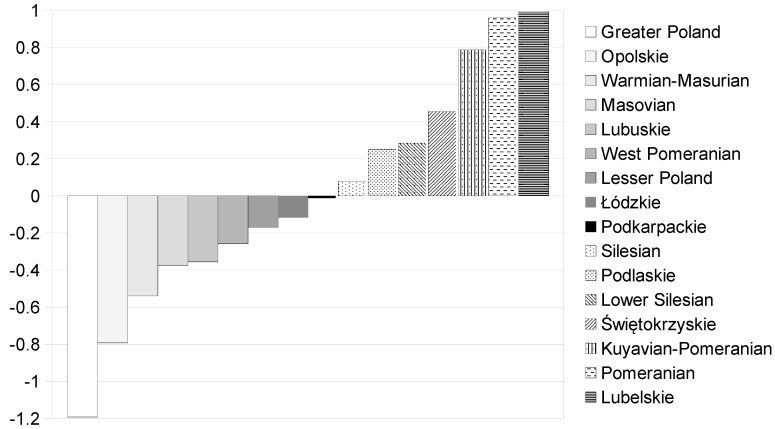
Comparison of Polish voivodeships with respect to the increasing value of the W^A^ indicator. Source: Authors’ calculations.

According to the classification of the W^A^ indicator value based on its standard deviation (see Table 4), we found that in three voivodeships (Kuyavian-Pomeranian, Pomeranian, and Lubelskie), the values were more than average, however, in two others (Greater Poland and Opolskie), they were below average. This means that cardiology rehabilitation facilities (regardless of local differences in ACS incidence) could be perceived as good in three voivodeships and insufficient in the two remaining districts, respectively. The remaining eleven voivodeships reached the average value of the W^A^ indicator, which could mean a sufficient number of rehabilitation facilities. Ranking of voivodeships with respect to the increasing value of the W^A^ indicator is presented at Figure 1.

**Figure 2:**
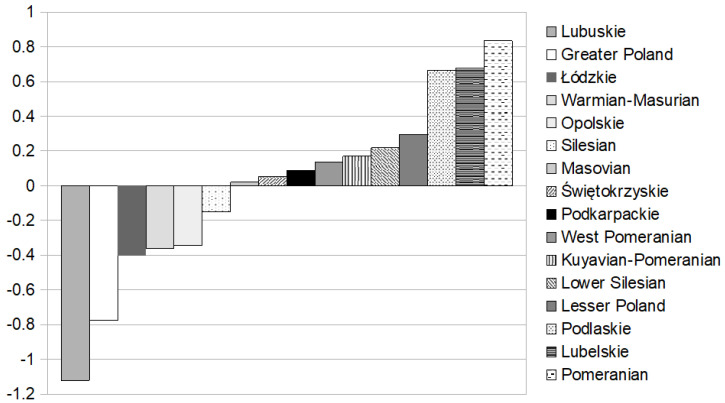
Comparison of Polish voivodeships with respect to the increasing value of the W^B^ indicator. Source: Authors’ calculations.

The value of the W^B^ indicator (additionally involving “de-stimulant” variable: the number of ACS in order to include differences in the ACS incidence among examined districts) was between −1.121 for Lubuskie and 0.834 for the Pomeranian voivodeships (see Table 4). The standard deviation of the W^B^ indicator was lower than W^A^ and gained 0.505, which showed a lower differentiation in the W^B^ value than W^A^. The ranking of voivodeships with respect to the W^B^ indicator value changed little. Three voivodeships obtained a more than average value of W^B^: Pomeranian, Lubelskie, and Podlaskie (the new one in this group of districts where the accessibility to cardiology rehabilitation facilities was the highest). The W^B^ indicator was below average for two voivodeships: Greater Poland and Lubuskie (the new one among districts where the access to rehabilitation could be described as lower than sufficient). The remaining eleven voivodeships reached the average value of the W^B^ indicator, which could mean sufficient accessibility to cardiology rehabilitation after ACS. Figure 2 shows the comparison of voivodeships with respect to the W^B^ indicator value in increasing order.

## 4. Discussion

Cardiological rehabilitation is an important part of circulatory system disease prevention and treatment [59,60]. Therefore, the issue of the evaluation of rehabilitation facilities has arisen. In other research, Perkal’s indicator, based on synthetic features aggregation, has been used for the assessment of the territorial diversification of services including healthcare [61,62]. The framework of this indicator allowed us to include both variables positively affecting the examined phenomena (as “stimulants”) and negatively (as “de-stimulants”). In our research, we used Perkal’s indicator in order to evaluate accessibility to cardiology rehabilitation after ACS in Poland. Two types of this synthetic indicator were employed. The first, called W^A^ in this paper, was composed of two “stimulants” describing the rehabilitation infrastructure (numbers of indoor and outdoor rehabilitation centers in each Polish voivodeships). To enable comparisons among voivodeships, “stimulants” were expressed per 100,000 population.

The W^A^ indicator allowed us to evaluate the differences in the equipment of Polish voivodeships with rehabilitation infrastructure. However, it disregards the demand for cardiology rehabilitation at the level of districts. The second type of Perkal’s indicator (called W^B^ in this paper), except for two of the above-mentioned “stimulants”, consisted of the “de-stimulant” variable: the number of ACS per 100,000 population. This variable could be perceived as an estimator of demand for rehabilitation after ACS. The use of “de-stimulants” in the construction of Perkal’s indicator has been previously applied by other authors [58,62].

There is an essential difference in relevance between indicators W^A^ and W^B^. The first of them reflects the potential ability of the healthcare system to provide rehabilitation services for patients in the early period after ACS. However, access to cardiological rehabilitation depends not only on the supply guaranteed by existing rehabilitation centers, but also by the demand created by the number of cases of ACS. The W^A^ indicator securely evaluates the supply side of the rehabilitation process because it aggregates data on two different ways of rehabilitation: inpatient and outpatient. Assessment of demand by W^A^ is nevertheless rather weak, as the only one estimator of demand employed here was the size of the population of each voivodeship (numbers of rehabilitation centers are expressed per 100,000 population). It could be efficient approach only in the case of similar incidence of ACS among all voivodeships (which might be a false assumption). Better evaluation of cardiological rehabilitation provides the W^B^ indicator. This indicator contains a good estimator of demand: the number of ACS that allows assess not only to rehabilitation infrastructure, but also the real accessibility to rehabilitation services with regard to the need for rehabilitation after ACS.

The distinct relevance of W^A^ and W^B^ indicators was visible in the results of our investigations. Although the vast majority of examined Polish voivodeships reached at least an average value of Perkal’s indicators (both W^A^ and W^B^) that could identify sufficient development of a rehabilitation infrastructure, there were differences between the rankings of voivodeships with respect to the W^A^ and W^B^ values. Voivodeships better equipped with rehabilitation centers were favored in the W^A^ indicator ranking, while in the W^B^ ranking, there was awarded accessibility to existing rehabilitation centers taking into account not only the number of them, but also the patients’ needs expressed as ACS incidence. In light of this, the W^A^ indicator can be perceived as a measure of nominal capability of rehabilitation infrastructure, while the W^B^ indicator as a measure of the real availability of cardiological rehabilitation after ACS. A negative health variable as a de-stimulant in a design for an aggregated synthetic indicator has been used in earlier research [63].

Additionally, the standard deviation of the W^B^ indicator was lower than in the case of the W^A^. This is not only an evidence of lower differentiation of the W^B^ value than W^A^, but could also indicate a better match of the rehabilitation infrastructure with the real needs defined by ACS incidence than with only the population size of the voivodeships.

Further research with the usage of more data on the number of workers in the rehabilitation centers is reasonable in order to precisely identify the efficiency within the cardiological rehabilitation infrastructure. However, it would require more detailed databases regarding the staff of cardiology rehabilitation centers. This implies that policy makers should create a more detailed database. The obtained results are in line with the world research on the recognized barriers of geographic nature to access to CR (i.e., [40]).

As the voivodeships in Poland differ in their accessibility to cardiology rehabilitation centers, this means that some voivodeships were not implementing effective health policies, which would lead to improved access to cardiological rehabilitation services for people in their area. The analysis is particularly relevant for decision-makers and the results of this research should be taken into account in the process of regional planning and by healthcare sector decision-makers. It is of special importance as the proper establishment and development of CR services is essential for the most effective management of heart conditions, otherwise it would have a negative impact on the outcomes of implemented cardiological programs in Poland. Thus, the results presented in this article are important to continue the advancement of knowledge on the subject of equity in healthcare resource distribution and its impact on health.

## 5. Conclusions

On the basis of Perkal’s synthetic indicator, the accessibility to cardiology rehabilitation after acute coronary syndromes (ACS) in Poland was assessed. This is of high importance as cardiological rehabilitation is an important part of circulatory system disease prevention and treatment.

In this research, two types of this synthetic indicator were employed. The first one enabled us to evaluate differences in the equipment of Polish voivodeships with rehabilitation infrastructure. The second type of Perkal’s indicator allowed us to estimate the real availability of cardiological rehabilitation, considering the demand for rehabilitation after ACS, as access to rehabilitation depends not only on supply guaranteed by existing rehabilitation centers, but also by the needs created by the number of cases of ACS. The results showed that most voivodeships were sufficiently developed in terms of their rehabilitation infrastructure, however, there were some that suffered from a lower level of infrastructure. However, through a comparison of both Perkal’s indicators, we found a better match between the rehabilitation infrastructure and the real needs defined by ACS incidence than with only the population size of the voivodeships.

Hence, this study can be used as a basis for healthcare policy formulation in order to correct some inequalities of cardiological rehabilitation infrastructure. By having such information, the national government could monitor the nationwide distribution of cardiological rehabilitation infrastructure and provide some advice to regional policy makers (including National Health Funds) in order to make proper adjustments to the real demand for such services.

## Figures and Tables

**Table 1:** Number of cardiological rehabilitation centers and number of acute coronary syndromes (ACS) in Polish districts (voivodeships) in 2019.

District (Voivodeship)	Population (2018) ^a^	Number of Inpatient Rehab. Centr. ^b^	Number of Outpatient Rehab. Centr. ^b^	Total Number of Rehab. Centr.	Number of ACS ^c^
Lower Silesian	2,901,225	9	6	15	6811
Kuyavian-Pomeranian	2,077,775	5	8	13	5830
Lubelskie	2,117,619	7	7	14	5010
Lubuskie	1,014,548	2	2	4	3489
Łódzkie	2,466,322	4	7	11	6831
Lesser Poland	3,400,577	3	12	15	6429
Masovian	5,403,412	12	9	21	11,120
Opolskie	986,506	2	1	3	2131
Podkarpackie	2,129,015	3	7	10	4830
Podlaskie	1,181,533	4	2	6	2111
Pomeranian	2,333,523	11	4	15	5013
Silesian	4,533,565	8	14	22	11,889
Świętokrzyskie	1,241,546	2	5	7	3329
Warmian-Masurian	1,428,983	4	1	5	3403
Greater Poland	3,493,969	4	4	8	8232
West Pomeranian	1,701,030	4	3	7	3419
Total-Poland	38,411,148	84	92	176	89,877

Source: ^a^ Statistics Poland (Główny Urząd Statystyczny) 2018. https://stat.gov.pl; ^b^ Database of Polish National Health Fund 2019. https://zip.nfz.gov.pl/GSL/GSL/Szpitale (accessed on 17 November–8 December 2019), https://zip.nfz.gov.pl/GSL/GSL/PrzychodnieSpecjalistyczne (accessed on 8–15 December 2019); ^c^ Database of Polish National Health Fund 2019. https://statystyki.nfz.gov.pl (accessed on 22–29 December 2019).

**Table 2:** Statistics of variables used in the research calculated per 100,000 population.

k	District (Voivodeship)	Number of Inpatient Rehab. Cent. Per 100,000 Population X_1_	Number of Outpatient Rehab. Cent. Per 100,000 Population X_2_	Number of ACS Per 100,000 Population X_3_
1	Lower Silesian	0.31	0.21	234.76
2	Kuyavian-Pomeranian	0.24	0.39	280.59
3	Lubelskie	0.33	0.33	236.59
4	Lubuskie	0.20	0.20	343.90
5	Łódzkie	0.16	0.28	276.97
6	Lesser Poland	0.09	0.35	189.06
7	Masovian	0.22	0.17	205.80
8	Opolskie	0.20	0.10	216.01
9	Podkarpackie	0.14	0.33	226.87
10	Podlaskie	0.34	0.17	178.67
11	Pomeranian	0.47	0.17	214.83
12	Silesian	0.18	0.31	262.24
13	Świętokrzyskie	0.16	0.40	268.13
14	Warmian-Masurian	0.28	0.07	238.14
15	Greater Poland	0.11	0.11	235.61
16	West Pomeranian	0.24	0.18	201.00
Mean value		0.23	0.24	238.07
Standard deviation		0.10	0.10	39.94
Class of variable		stimulant	stimulant	de-stimulant

Source: Authors’ calculation.

**Table 3:** Normalized values of variables used in the research.

k	District (Voivodeship)	Normalized Value of Inpatient Rehab. Centers Z_1_	Normalized Value of Outpatient Rehab. Centers Z_2_	Normalized Value of ACS Z_3_
1	Lower Silesian	0.849	−0.278	0.083
2	Kuyavian-Pomeranian	0.117	1.458	−1.064
3	Lubelskie	1.063	0.927	0.037
4	Lubuskie	−0.340	−0.373	−2.649
5	Łódzkie	−0.708	0.472	−0.974
6	Lesser Poland	−1.485	1.145	1.227
7	Masovian	−0.078	−0.670	0.808
8	Opolskie	−0.281	−1.305	0.552
9	Podkarpackie	−0.931	0.910	0.281
10	Podlaskie	1.147	−0.644	1.487
11	Pomeranian	2.544	−0.623	0.582
12	Silesian	−0.558	0.715	−0.605
13	Świętokrzyskie	−0.719	1.630	−0.753
14	Warmian-Masurian	0.530	−1.611	−0.002
15	Greater Poland	−1.209	−1.178	0.062
16	West Pomeranian	0.060	−0.575	0.928

Source: Authors’ calculation.

**Table 4:** Values and classification of Perkal’s indicators (W^A^ and W^B^).

District (Voivodeship)	W^A^ Indicator	Class of W^A^	W^B^ Indicator	Class of W^B^
Lower Silesian	0.285	average	0.218	average
Kuyavian-Pomeranian	0.788	above average	0.170	average
Lubelskie	0.995	above average	0.676	above average
Lubuskie	−0.356	average	−1.121	below average
Łódzkie	−0.118	average	−0.403	average
Lesser Poland	−0.170	average	0.295	average
Masovian	−0.374	average	0.020	average
Opolskie	−0.793	below average	−0.345	average
Podkarpackie	−0.011	average	0.086	average
Podlaskie	0.251	average	0.663	above average
Pomeranian	0.960	above average	0.834	above average
Silesian	0.079	average	−0.149	average
Świętokrzyskie	0.455	average	0.053	average
Warmian-Masurian	−0.540	average	−0.361	average
Greater Poland	−1.193	below average	−0.775	below average
West Pomeranian	−0.258	average	0.138	average
Standard deviation	0.592		0.505	
Classification of WA/WB indicators value	>0.592 ≥−0.592; ≤0.592 <−0.592	above average average below average	>0.505 ≥−0.505; ≤0.505 <−0.505	above average average below average

Source: Authors’ calculations.
